# *EPAS1* is associated with human muscle fiber composition and endurance phenotypes

**DOI:** 10.14814/phy2.70928

**Published:** 2026-05-19

**Authors:** Albina Z. Dautova, Elena V. Valeeva, Ekaterina A. Semenova, Fanis A. Mavliev, Alexey A. Zverev, Andrey S. Nazarenko, George John, Andrey V. Zhelankin, Andrey K. Larin, Nikolay A. Kulemin, Rinat I. Sultanov, Edward V. Generozov, Ildus I. Ahmetov

**Affiliations:** ^1^ Research Institute of Physical Culture and Sport Volga Region State University of Physical Culture, Sport and Tourism Kazan Russia; ^2^ Laboratory of Genetics of Aging and Longevity Kazan State Medical University Kazan Russia; ^3^ Transform Specialist Medical Centre Dubai UAE; ^4^ Department of Molecular Biology and Genetics Lopukhin Federal Research and Clinical Center of Physical‐Chemical Medicine of Federal Medical Biological Agency Moscow Russia; ^5^ Sports Genetics Laboratory St Petersburg Research Institute of Physical Culture St Petersburg Russia; ^6^ Research Institute for Sport and Exercise Sciences Liverpool John Moores University Liverpool UK

**Keywords:** aerobic performance, athletic performance, genotype, muscle, polymorphism, talent

## Abstract

A recent study reported that endothelial PAS domain protein 1 (EPAS1; hypoxia‐inducible factor 2α) acts downstream of PGC‐1α to regulate the slow‐twitch muscle fiber program in mice, yet its role in human physiology and exercise adaptation remains unclear. The aim of this study was threefold: (1) to investigate *EPAS1* gene expression in human skeletal muscle and its association with muscle fiber composition and the expression of endurance‐related genes; (2) to determine how *EPAS1* expression responds to aerobic training; and (3) to examine whether *EPAS1* genetic variation is linked to aerobic capacity, hemoglobin, and athletic status. The study involved 1234 subjects, including 943 athletes and 291 untrained individuals. *EPAS1* gene expression was significantly higher in endurance athletes compared with power athletes (*p* = 0.011) and was positively associated with the proportion of slow‐twitch muscle fibers in the vastus lateralis of untrained subjects (*p* = 0.0008) and athletes (*p* = 0.0033). *EPAS1* expression was higher in females (*p* = 0.0028) and negatively associated with smoking status (*p* = 0.0007). Moreover, *EPAS1* expression showed positive association with endurance‐related genes, including *ANGPT2*, *CKM*, *CPT1B*, *EPOR*, *FNDC5*, *HIF1A*, *KDR*, *MYBPC3*, *NFATC4*, *NOS3*, *PPARA*, *PPARD*, *PPARGC1A*, *UCP2*, and *VEGFA*. Analysis of 24 publicly available skeletal muscle transcriptomic datasets demonstrated that *EPAS1* expression increased significantly (meta‐analysis *p* = 9.2 × 10^−5^) following aerobic training. Finally, genetically predicted higher *EPAS1* expression (i.e., carriage of the *EPAS1* rs6756667 A allele) was positively associated with endurance athlete status in both sexes (*p* = 0.0004) and with VO₂max (*p* = 0.046) and hemoglobin (*p* = 0.041) in male athletes. These findings potentially identify *EPAS1* as an important genetic factor associated with muscle fiber composition, endurance‐related phenotypes, and adaptation to aerobic training.

## 1 INTRODUCTION

The hypoxia that develops in skeletal muscle during intense exercise promotes adaptations that enhance oxygen delivery and utilization. In this context, particular attention has been given to genes regulating the activity of hypoxia‐inducible factor (HIF), an oxygen‐sensitive transcriptional complex (Semenza, 2011; Wang et al., 1995). HIF is a heterodimer consisting of one alpha subunit (HIFα) and one beta subunit (HIFβ). Several HIFα isoforms exist: HIF1α, HIF2α (also known as Endothelial PAS domain protein 1; EPAS1), and HIF3α, each with distinct biological properties (Majmundar et al., 2010). Three genes encode these isoforms in humans: *HIF1A*, *EPAS1*, and *HIF3A* (Greer et al., 2012; Makino et al., 2002; Wang et al., 1995).

EPAS1 activates gene expression in response to hypoxia (Patel & Simon, 2008). It plays a key role in catecholamine and mitochondrial homeostasis, cardiac output regulation, and erythropoietin synthesis (Formenti et al., 2011; Moon et al., 2022; Rankin et al., 2007; Tian et al., 1998). Prolonged bed rest has been shown to induce a significant reduction in *EPAS1* expression in human skeletal muscle, as demonstrated by genome‐wide transcriptomic analyses (Fernandez‐Gonzalo et al., 2020). In contrast, extreme endurance exercise is associated with enrichment of EPAS1 among transcription factors in blood, reflecting activation of hypoxia‐inducible pathways (Maqueda et al., 2017). Experimental studies in animal models have further shown that exhaustive exercise increases skeletal muscle HIF‐2α levels (Baker & Parise, 2016). In addition, a mechanistic study demonstrated that EPAS1 functions downstream of peroxisome proliferator‐activated receptor‐γ coactivator‐1α (PGC‐1α) to regulate the slow‐twitch muscle fiber programme in mice (Rasbach et al., 2010). Yet, its relevance to human physiology and exercise adaptation, as well as the coordination between *EPAS1* expression and other genes in human skeletal muscle, remains largely unclear. Skeletal muscle fiber composition, particularly the proportion of oxidative slow‐twitch (type I) fibers, is a key determinant of endurance performance, as these fibers exhibit greater mitochondrial density, fatigue resistance, and capacity for sustained aerobic metabolism (Hall et al., 2021; Schiaffino & Reggiani, 2011).

Under normoxic conditions, EPAS1 is targeted for proteasomal degradation by the tumor suppressor VHL (von Hippel–Lindau) (Ivan et al., 2001). In hypoxia, this degradation is inhibited, allowing the α subunit to dimerise with HIFβ. The resulting HIF complex binds to hypoxia‐response elements in promoters and enhancers of more than 100 hypoxia‐regulated genes, including erythropoietin (*EPO*), transferrin (*TF*), vascular endothelial growth factor (*VEGFA*), and glucose transporter 1 (*SLC2A1*), thereby improving oxygen delivery to tissues (Ke & Costa, 2006). Oxygen delivery to skeletal muscle during endurance exercise is considered one of the primary limiting factors for VO_2_max (Bassett Jr & Howley, 2000). Consequently, as both a detector of hypoxia and an activator of pathways that enhance tissue oxygenation, EPAS1 is a plausible candidate gene influencing endurance performance (Bondareva et al., 2016; Henderson et al., 2005).

The *EPAS1* gene is located on the short arm of chromosome 2 (2p21). It is expressed across all human tissues, with the highest levels observed in the lungs, vascular endothelial cells, reproductive organs, urinary system, adipose tissue, heart, skin, and the digestive tract (GTEx Portal, 2025). Several studies have shown that *EPAS1* polymorphisms are associated with erythropoietin and hemoglobin levels (Beall et al., 2010; Percy, 2008), traits that may in turn influence inter‐individual variation in athletic performance.

Consistent with its physiological relevance, genome‐wide analyses have identified exceptionally strong allele‐frequency differentiation near *EPAS1* in high‐altitude Tibetan populations (Beall et al., 2010). In particular, the A allele of rs6756667 in *EPAS1* is substantially more common in indigenous Tibetans than in lowland populations, with increasing altitude showing a linear decrease in the GG and AG genotypes and a corresponding rise in the AA genotype (Basang et al., 2015). According to the eQTLGen database (Võsa et al., 2021), the A allele of rs6756667 is strongly associated with increased *EPAS1* gene expression in blood (*z*‐score = 200.8; *p* = 3.1 × 10^−103^) in a cohort of 31,467 individuals, and can therefore be regarded as a robust predictor (proxy) of elevated *EPAS1* expression. The rs6756667 A allele of *EPAS1* has been associated with improved high‐altitude adaptation, showing a protective effect against altitude sickness and occurring at higher frequencies in mid‐ and high‐altitude populations than in lowland groups (Guo et al., 2015; Shen et al., 2020; Zhang et al., 2020).

Despite the well‐established role of *EPAS1* in high‐altitude adaptation, relatively few studies have investigated its polymorphisms in relation to elite athletic performance (Ahmetov et al., 2024; Bondareva et al., 2016; Bondareva & Godina, 2016; Henderson et al., 2005; Maciejewska‐Skrendo et al., 2019; Voisin et al., 2014), while only one pilot study has specifically examined rs6756667 in athletes, reporting a higher frequency of the A allele among 40 endurance‐trained athletes compared with 40 controls (Jiang et al., 2016).

The aim of this study was therefore threefold: (1) to investigate *EPAS1* gene expression in human skeletal muscle and its association with muscle fiber composition and the expression of endurance‐related genes; (2) to determine how *EPAS1* expression responds to aerobic training; and (3) to examine whether *EPAS1* genetic variation is linked to aerobic capacity, hemoglobin levels, and athletic status. We hypothesized that *EPAS1* expression in human skeletal muscle would be positively associated with the proportion of slow‐twitch muscle fibers and with the coordinated expression of multiple endurance‐related genes, would be higher in endurance‐trained athletes compared with power athletes, and would increase in response to aerobic training. We further hypothesized that genetically predicted higher *EPAS1* expression would be associated with endurance athlete status, higher aerobic capacity (VO_2_max), and elevated hemoglobin levels. In addition, we sought to determine whether *EPAS1* expression is influenced by sex and smoking status, given the known sex differences in muscle phenotype and the adverse effects of cigarette smoke on oxidative capacity. This hypothesis is supported by the known role of EPAS1 in hypoxia signaling, angiogenesis, mitochondrial function, and its downstream position relative to PGC‐1α in regulating slow‐twitch fiber programmes in animal models (Rasbach et al., 2010), as well as its established involvement in high‐altitude adaptation and erythropoiesis.

## 2 MATERIALS AND METHODS

### 2.1 Ethical approval

This study was approved by the Bioethical Committee of the Volga Region State University of Physical Culture, Sports and Tourism (reference #2; approval date: 26 May 2023) and the Ethics Committee of the Federal Research and Clinical Center of Physical–Chemical Medicine of the Federal Medical and Biological Agency of Russia (reference #2017/04; approval date: 4 July 2017). Written informed consent was obtained from all participants prior to the start of the study. For athletes younger than 18 years, consent was provided by their legal guardians in accordance with Russian legislation, which defines 18 years as the age of legal adulthood for biomedical research. The study was conducted in compliance with the Declaration of Helsinki and followed ethical standards for sport and exercise science research.

### 2.2 Overall study architecture and cohort descriptions

This study employed a multi‐level design to investigate the role of the *EPAS1* gene in muscle physiology and physical performance, integrating original experimental data with secondary analysis of large‐scale transcriptomic databases. The study architecture consisted of four distinct components:

1. Discovery and validation of gene expression: Original data from 24 male athletes (biopsy, immunohistochemistry, and transcriptomic‐based fiber‐type prediction) and secondary data from 291 untrained individuals (transcriptomic‐based fiber‐type prediction and expression of *EPAS1* and endurance‐related genes).
2. Training‐induced modulation: A meta‐analysis of 40 publicly available transcriptomic cohorts (*n* = 690) to assess *EPAS1* response to aerobic and resistance training.
3. Genetic case–control study: Original genotyping of 744 athletes to compare allele frequencies between endurance and power athletes.
4. Phenotypic associations: Evaluation of *EPAS1* genotype associations with VO_2_max (*n* = 160) and hemoglobin levels (*n* = 258) in athletes.

### 2.3 Participants

The study included 1234 participants: 943 athletes and 291 untrained individuals. Gene expression in the m. vastus lateralis was analyzed in 24 male athletes (GEO accession number GSE200398) and 291 untrained individuals (dbGaP accession number phs001048.v2.p1). The case–control study involved 398 endurance athletes and 346 power athletes. All athletes included in the genetic analyses were of Caucasian ancestry (Russians), which should be considered when interpreting allele‐frequency differences. Endurance athletes comprised rowers (*n* = 76), cross‐country skiers (*n* = 124), middle‐ and long‐distance runners (*n* = 30), biathletes (*n* = 32), road cyclists (*n* = 12), middle‐distance kayakers and canoeists (*n* = 25), 1.5–10 km speed skaters (*n* = 37), middle‐ and long‐distance swimmers (*n* = 27), race walkers (*n* = 7), orienteers (*n* = 5), and triathletes (*n* = 23). Power athletes included 100–400 m runners (*n* = 21), short‐distance cyclists (*n* = 32), short‐distance kayakers and canoeists (*n* = 29), decathletes (*n* = 4), 500–1000 m speed skaters (*n* = 40), throwers (*n* = 10), powerlifters (*n* = 34), 50–100 m swimmers (*n* = 66), jumpers (*n* = 35), climbers (*n* = 7), skeleton athletes (*n* = 3), artistic gymnasts (*n* = 1), weightlifters (*n* = 61), and 500–100 m short‐track speed skaters (*n* = 3).

Hematological parameters were evaluated in 258 athletes (110 females and 148 males), of whom 100 were under 18 years of age at the time of participation. The cohort comprised athletes from the following sports: swimming (*n* = 52), rowing (*n* = 18), cross‐country skiing (*n* = 16), triathlon (*n* = 9), biathlon (*n* = 9), orienteering (*n* = 5), cycling (*n* = 5), running (*n* = 12), speed skating (*n* = 6), short‐track speed skating (*n* = 3), kayaking (*n* = 10), canoeing (*n* = 5), wrestling (*n* = 22), boxing (*n* = 8), figure skating (*n* = 12), ice hockey (*n* = 11), tennis (*n* = 9), table tennis (*n* = 7), baseball (*n* = 7), badminton (*n* = 6), volleyball (*n* = 4), water polo (*n* = 2), basketball (*n* = 1), alpine skiing (*n* = 3), sailing (*n* = 1), ski jumping (*n* = 1), modern pentathlon (*n* = 1), weightlifting (*n* = 8), and artistic gymnastics (*n* = 4).

VO_2_max was assessed in 160 athletes (55 females and 105 males), of whom 82 were under 18 years of age at the time of participation. The cohort comprised athletes from the following sports: rowing (*n* = 21), basketball (*n* = 10), athletics (*n* = 10), swimming (*n* = 38), cross‐country skiing (*n* = 29), football (*n* = 18), tennis (*n* = 13), table tennis (*n* = 3), triathlon (*n* = 7), wrestling (*n* = 5), orienteering (*n* = 3), boxing (*n* = 2), and canoeing (*n* = 1).

Athletes represented all performance levels, including non‐elite (*n* = 205), sub‐elite (*n* = 129), elite (*n* = 271), and highly elite (*n* = 338). The general characteristics of the studied groups are presented in Table 1. Additionally, skeletal muscle *EPAS1* gene expression was analyzed following aerobic training of at least 3 weeks using 24 publicly available datasets (381 individuals) and following resistance training using 16 cohorts (309 individuals) (MetaMEx, 2025).

**TABLE 1:** General characteristics of the studied groups.

Study	Group	Sex	*n*	Age, years	Height, cm	Body mass, kg
Gene expression 1	Untrained	Females	125	60.3 (8.1)	162.8 (5.6)	71.8 (9.8)
	Untrained	Males	166	59.5 (8.1)	176.7 (6.7)	87.3 (15.1)
Gene expression 2	Power athletes	Males	10	30.1 (7.4)	178.2 (6.7)	85.6 (12.4)
	Endurance athletes	Males	14	34.6 (9.7)	182.6 (6.6)	76.1 (9.5)
Case–control study	Power athletes	Females	137	25.1 (5.7)	170.1 (8.0)	63.6 (10.3)
	Power athletes	Males	209	25.7 (6.0)	181.5 (7.7)	85.1 (18.1)
	Endurance athletes	Females	169	25.8 (4.7)	168.8 (7.2)	59.9 (7.7)
	Endurance athletes	Males	229	26.0 (6.2)	183.2 (8.8)	77.9 (13.2)
Hematological study	Athletes	Females	110	20.9 (3.2)	169.2 (8.2)	63.7 (7.2)
	Athletes	Males	148	20.2 (4.7)	183.6 (10.4)	81.2 (12.2)
VO_2_max study	Athletes	Females	55	21.3 (7.9)	166.1 (7.8)	57.4 (11.2)
	Athletes	Males	105	22.5 (7.5)	179.4 (11.9)	70.9 (16.0)

*Note*: Data are mean (SD).

### 2.4 Genotyping

DNA samples from 766 athletes were genotyped using microarray technology, as previously described (Moreland et al., 2022). Briefly, DNA was extracted from leukocytes obtained from venous blood. Four milliliters of venous blood were collected into EDTA‐containing tubes (Vacuette EDTA tubes; Greiner Bio‐One, Kremsmünster, Austria). Samples were transported to the laboratory at 4°C and processed on the same day. DNA extraction and purification were carried out using a commercial kit Techno‐Sorb (Technoclon, Moscow, Russia) according to the manufacturer's instructions. The assay required 200 ng of DNA with a minimum concentration of 50 ng/μL. DNA concentrations were quantified using a Qubit Fluorometer (Invitrogen, Waltham, MA, USA). Genotyping was conducted using HumanOmni1‐Quad BeadChips (Illumina, San Diego, CA, USA), covering 1,140,419 SNPs, including *EPAS1* rs6756667. All subsequent procedures were performed following the Infinium High‐Density Assay protocol. Ten percent of the samples were re‐genotyped, yielding a 100% reproducibility rate. DNA samples from an additional 177 athletes were isolated from buccal epithelial cells collected using a sterile disposable scraping probe. DNA was extracted using the sorbent method in accordance with the instructions for the AmpliSens DNA‐sorb‐B kit (ILS, Moscow, Russia; catalogue number K1‐2‐100‐CE). These samples were genotyped by real‐time PCR using a CFX96 Touch system (Bio‐Rad, USA) with custom‐designed assay reagents (TestGen, Ulyanovsk, Russia).

### 2.5 Gene expression analysis

For the athlete cohort, athletes were instructed to refrain from training for 1 day prior to biopsy of the vastus lateralis in the left leg, allowing analysis of gene expression profiles in the resting state (Semenova et al., 2022). Biopsies were collected during the athletes' regular training period, and although training volumes and intensities varied between individuals and between endurance and power subgroups, all participants were engaged in consistent sport‐specific training multiple times per week. Immediately after collection, muscle tissue samples were snap‐frozen in liquid nitrogen to preserve RNA integrity and subsequently stored at −80°C until further processing. RNA was extracted from 24 muscle tissue samples using the RNeasy Mini Fibrous Tissue Kit (Qiagen, Venlo, The Netherlands; catalogue number 74704). Each sample was then transferred onto a sterile Petri dish positioned on a frozen plastic ice pack. Approximately 10 mg of tissue was excised using a sterile scalpel and immediately placed into a 2 mL safe‐lock microcentrifuge tube containing 300 μL of lysis buffer and a sterile 4 mm stainless‐steel bead. Samples were homogenized in the TissueLyser II system (Qiagen, Venlo, The Netherlands; catalogue number 85300) with two 2‐min cycles at 25 Hz. RNA isolation followed the manufacturer's protocol. Concentration of RNA was measured using the Qubit spectrophotometer (Thermo Fisher Scientific, Waltham, MA, USA), and RNA integrity was evaluated with the BioAnalyzer electrophoresis system employing the BioAnalyzer RNA Nano assay (Agilent Technologies, Santa Clara, CA, USA). RNA integrity numbers (RIN) were calculated, and only samples with RIN > 7 were retained for further analysis. Total RNA underwent DNase I treatment using the Turbo DNA‐free Kit (Thermo Fisher Scientific, Waltham, MA, USA; catalogue number AM1907) according to manufacturer instructions. Quality control of sequencing reads was performed using FastQC and MultiQC both before and after adapter trimming with Cutadapt and quality filtering with Trimmomatic. Samples were stored at −80°C until library preparation. RNA‐seq libraries were prepared using the Illumina NEBNext Ultra II Directional RNA Library Prep Kit (New England Biolabs, Ipswich, MA, USA; catalogue number E7760) with the NEBNext rRNA Depletion Module (New England Biolabs, Ipswich, MA, USA; catalogue number E7400) and sequenced on the Illumina HiSeq platform with 250 cycles. Sequenced reads were pseudoaligned to the hg38 GENCODE (v37) transcriptome using kallisto v0.48.0 (Bray et al., 2016) with default settings. Gene‐level expression estimates were generated using the tximport Bioconductor package (Soneson et al., 2015) and are presented as transcripts per million (TPM).

For the untrained cohort (*n* = 291), derived from publicly available datasets, RNA isolation and sequencing were conducted using strand‐specific mRNA‐seq as described previously (Taylor et al., 2019). Because these individuals were not subjected to experimental training protocols, no training restriction applied to this cohort. Gene fragments were counted using htseq‐count v0.5.4 (Anders et al., 2015) based on GENCODE v19 annotations (Harrow et al., 2012), and expression of *EPAS1* and endurance‐related genes (*AMPD1*, *ANGPT1*, *ANGPT2*, *CKM*, *CPT1B*, *EPOR*, *ESRRA*, *ESRRG*, *FNDC5*, *GABPB1*, *HIF1A*, *KDR*, *MYBPC3*, *NFATC4*, *NOS3*, *PPARA*, *PPARD*, *PPARGC1A*, *PPARGC1B*, *PRKAA1*, *PRKAA2*, *TFAM*, *UCP2*, *UCP3*, and *VEGFA*) was quantified in TPM. These genes were selected a priori based on strong experimental and epidemiological evidence from genome‐wide association studies, transcriptomic analyses, and functional studies in both human and animal models supporting their involvement in key physiological pathways underlying endurance performance, including mitochondrial biogenesis, oxidative metabolism, angiogenesis, oxygen transport, muscle fiber specification, and hypoxia‐responsive signaling (Table S1).

Smoking status was recorded as a binary variable (0 = non‐smoker and 1 = current smoker) and included as a covariate in all regression models because cigarette smoke is a known suppressor of hypoxia‐inducible signaling pathways and skeletal muscle oxidative capacity.

### 2.6 VO_2max_ measurement

Aerobic capacity in rowers, swimmers, and cross‐country skiers was evaluated using a graded exercise test with stepwise increases in workload on a PM3 mechanical rowing ergometer (Concept II, USA). The initial workload was set at 150 W, with each stage lasting 3 min, a 30 s rest interval between stages, and a workload increment of 50 W at each subsequent stage. In athletes from other sports (basketball, athletics, football, tennis, table tennis, triathlon, wrestling, orienteering, boxing, and canoe), aerobic capacity was assessed using an incremental exercise test on a Saturn treadmill (HP Cosmos, Germany), starting at a speed of 7 km·h^−1^ with increases of 0.1 km·h^−1^ every 10 s until volitional exhaustion. Respiratory gas exchange and heart rate were recorded breath‐by‐breath throughout the test using a MetaLyzer 3B gas analyzer (Cortex, Germany). Absolute and relative maximal oxygen uptake (VO_2_max) were determined as the mean value over the final 30 s of exercise.

### 2.7 Evaluation of muscle fiber composition

In 24 male athletes, vastus lateralis biopsies were collected from the left leg using a modified Bergström needle technique with suction, under local anesthesia with a 2% lidocaine solution. Immediately after collection, samples were snap‐frozen in liquid nitrogen and stored at −80°C until further processing. Frozen specimens were sectioned serially (7 μm thickness) using an ultramicrotome (Leica Microsystems, Wetzlar, Germany). Sections were thaw‐mounted onto Polysine‐coated glass slides, left at room temperature (RT) for 15 min, and washed in phosphate‐buffered saline (PBS; 3 × 5 min). Subsequently, sections were incubated at RT for 1 h with primary antibodies directed against slow and fast myosin heavy chain isoforms (M8421, 1:5000; M4276, 1:600, respectively; Sigma‐Aldrich, St. Louis, MO, USA), followed by rinsing in PBS (3 × 5 min). The sections were then exposed for 1 h at RT to FITC‐conjugated secondary antibodies (F0257; 1:100; Sigma‐Aldrich, St. Louis, MO, USA). After antibody removal, slides were washed in PBS (3 × 5 min), mounted in appropriate mounting medium, and sealed with a coverslip. Fluorescence images were acquired using an Eclipse Ti‐U microscope (Nikon, Tokyo, Japan). Each analyzed image comprised 334 ± 14 muscle fibers. Fiber‐type proportions were determined as the ratio of immunostained fibers to the total number of fibers.

In 291 untrained individuals, muscle samples were obtained from the vastus lateralis using a conchotome under local anesthesia with 20 mg·mL^−1^ lidocaine hydrochloride without epinephrine. Muscle fiber composition was estimated based on the expression of myosin heavy chain 1 (*MYH1*), myosin heavy chain 2 (*MYH2*), and myosin heavy chain 7 (*MYH7*) genes, as previously described (Taylor et al., 2019). Briefly, it was assumed that the TPM count of each gene is proportional to the relative abundance of the corresponding fiber type. To estimate fiber‐type proportions, the expression (TPM) of each gene was divided by the sum of the TPMs of all three genes. To validate the transcriptomic approach for estimating fiber‐type composition, we compared the predicted slow‐twitch fiber proportions with those obtained by immunohistochemistry in the same 24 male athletes.

### 2.8 Hematological analysis

Blood samples were collected from the athletes in the morning after an overnight fast, 1 day after the training session. Hemoglobin levels were measured using an automated hematology analyzer (MEK‐7222K, Nihon Kohden, Japan).

### 2.9 Statistical analyses

Statistical analyses were performed using GraphPad InStat software (version 3.05; GraphPad Software, Inc., CA, USA). Genotype distributions and allele frequencies between athletes and controls were compared using χ^2^ tests with Bonferroni correction for multiple comparisons. Between‐group differences in phenotypic variables (gene expression) were analyzed using unpaired *t*‐tests. The relationships between *EPAS1* gene expression, muscle fiber composition, and the expression of endurance‐related genes were examined using multiple regression analyses adjusted for age, sex, and smoking status, with smoking status coded as a binary variable (0 = non‐smoker and 1 = current smoker). As *EPAS1* was analyzed as a priori candidate gene, genome‐wide differential expression methods were not applied. Associations between the *EPAS1* polymorphism, VO_2_max, and hemoglobin levels were assessed using multiple regression models adjusted for age, sex, training type, and level of athletic achievement. β values represent unstandardized regression coefficients. The correlation between transcriptomically predicted and immunohistochemically measured fiber‐type proportions was assessed using Pearson's correlation coefficient. A meta‐analysis of 24 publicly available skeletal muscle transcriptomic datasets was performed, as previously described (Pillon et al., 2020). Data are presented as mean (standard deviation). A *p* value <0.05 was considered statistically significant.

## 3 RESULTS

### 3.1 *EPAS1* gene expression in relation to muscle fiber composition and endurance‐related genes: Evidence from primary athlete data and secondary database analyses

**FIGURE 1:**
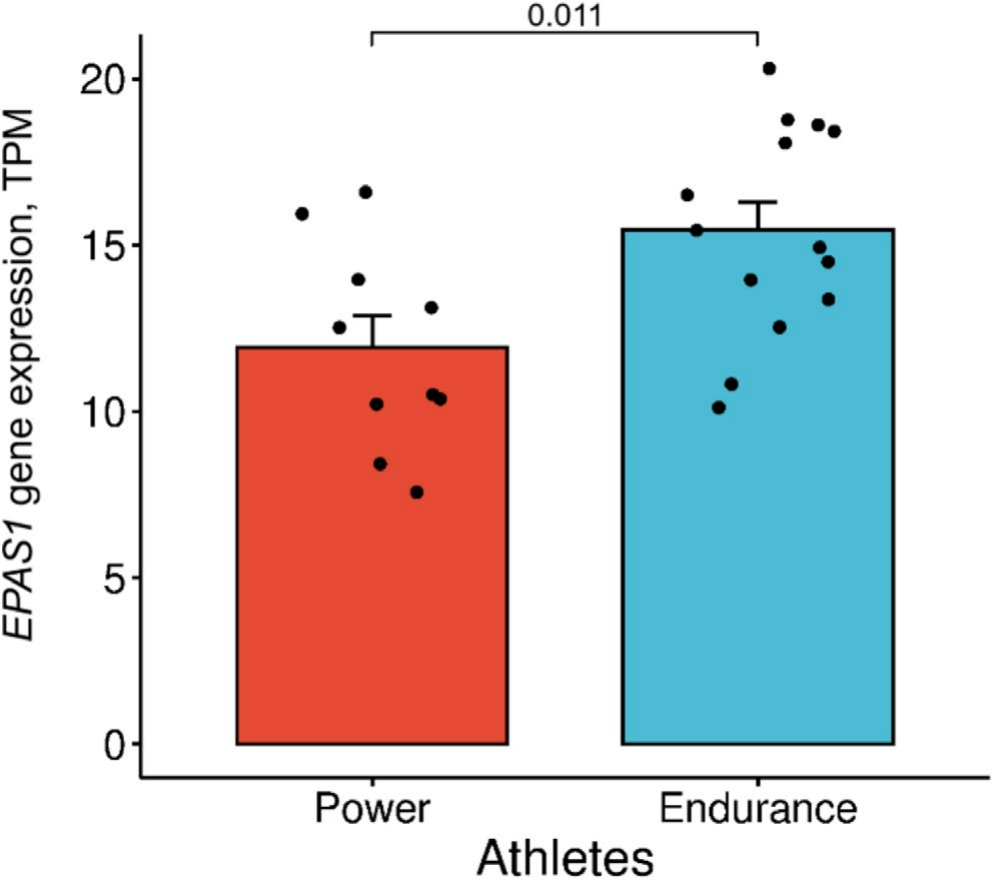
*EPAS1* gene expression in the vastus lateralis of endurance and power athletes (*p* = 0.011).

In male athletes (*n* = 24), *EPAS1* gene expression was significantly higher in the vastus lateralis of endurance compared with power athletes (15.5 ± 3.1 vs. 11.9 ± 3.0 TPM; *p* = 0.011) (Figure 1).

To validate the use of transcriptomically predicted fiber‐type proportions, we first applied the approach of Taylor et al. (2019), which estimates slow‐ and fast‐twitch fiber proportions based on the expression of muscle fiber‐specific genes, in this cohort of 24 male athletes. Predicted proportions showed a strong correlation with actual fiber‐type composition assessed by immunohistochemistry (Pearson *r* = 0.92, *p* = 3.8 × 10^−10^), confirming the reliability of this proxy method.

**FIGURE 2:**
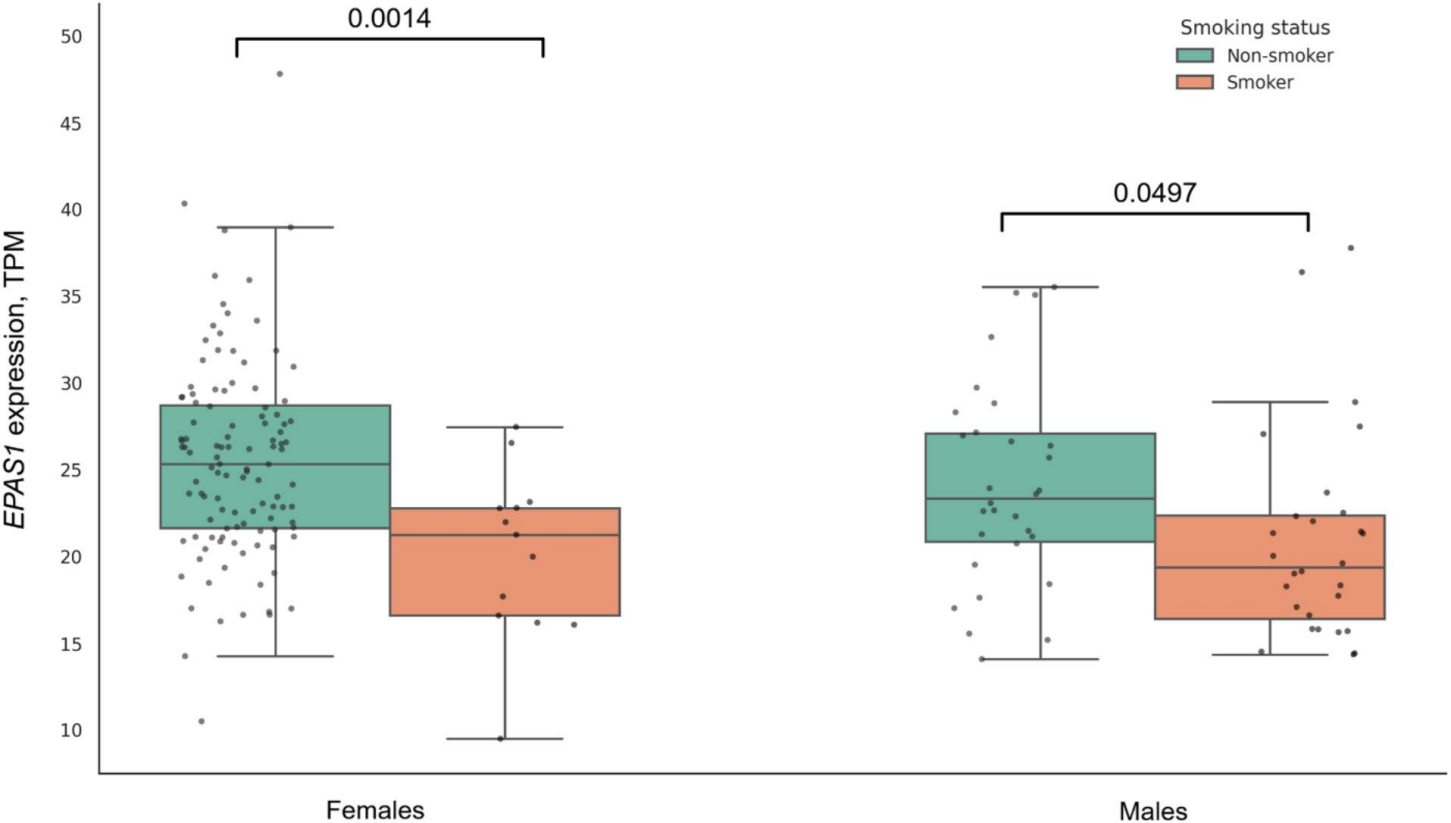
*EPAS1* gene expression by smoking status in males and females.

Having validated the prediction method, we then examined the association between slow‐twitch fiber proportion and *EPAS1* expression in a larger cohort of untrained individuals, for whom only transcriptomically predicted fiber‐type data were available. In this untrained group, *EPAS1* expression was higher in females than in males (25.0 (5.9) vs. 22.8 (5.6) TPM; *p* = 0.0028) and negatively associated with smoking status after adjusting for age and sex (*β* = −3.35; *p* = 0.0007). Stratified analyses by sex showed that male non‐smokers had higher *EPAS1* expression compared with smokers (23.1 (5.5) vs. 20.9 (6.0) TPM; *p* = 0.0497), whereas female non‐smokers also exhibited increased expression relative to smokers (25.5 (5.7) vs. 20.1 (4.9) TPM; *p* = 0.0014) (Figure 2). This analysis was performed to test whether an environmental factor known to impair oxidative capacity (smoking) modulates *EPAS1* expression in human skeletal muscle, thereby extending the central hypothesis of the study.

**FIGURE 3:**
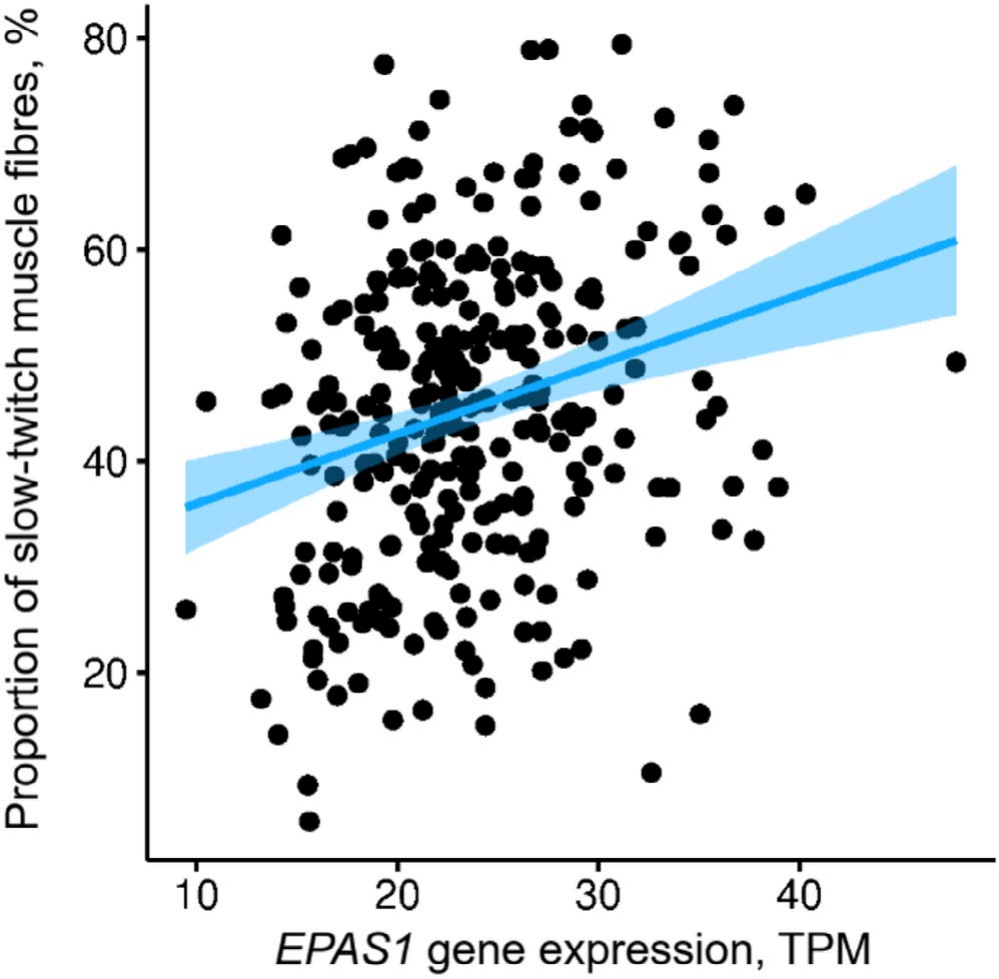
Moderate positive association between *EPAS1* gene expression and the predicted proportion of slow‐twitch muscle fibers in untrained individuals (*β* = 0.486; *p* = 0.0008 after adjustment for age, sex, and smoking status). Considerable scatter is evident, consistent with the limitations of transcriptomic fiber‐type estimation.

Furthermore, *EPAS1* expression showed a moderate positive association with the predicted proportion of slow‐twitch fibers in the vastus lateralis after adjusting for age, sex, and smoking status (*β* = 0.486; *p* = 0.0008) (Figure 3). Although statistically significant, the association exhibited considerable scatter, consistent with the known limitations of the transcriptomic proxy for fiber‐type composition. This association was replicated in the independent cohort of 24 male athletes (*β* = 3.04; *p* = 0.0033) after adjusting for age.

Finally, *EPAS1* expression showed positive associations with multiple endurance‐related genes, including *ANGPT2* (*β* = 4.3; *p* < 0.0001), *CKM* (*β* = 0.0003; *p* < 0.0001), *CPT1B* (*β* = 8.6; *p* = 0.0001), *EPOR* (*β* = 11.0; *p* < 0.0001), *FNDC5* (*β* = 0.05; *p* = 0.0005), *HIF1A* (*β* = 3.6; *p* < 0.0001), *KDR* (*β* = 2.8; *p* < 0.0001), *MYBPC3* (*β* = 26.5; *p* < 0.0001), *NFATC4* (*β* = 6.0; *p* < 0.0001), *NOS3* (*β* = 8.3; *p* < 0.0001), *PPARA* (*β* = 0.5; *p* = 0.0276), *PPARD* (*β* = 1.1; *p* < 0.0001), *PPARGC1A* (*β* = 0.05; *p* = 0.0089), *UCP2* (*β* = 0.11; *p* = 0.0397), and *VEGFA* (*β* = 0.5; *p* < 0.0001), after adjustment for age, sex, and smoking status. Conversely, no significant associations were observed between *EPAS1* expression and the expression of *AMPD1*, *ANGPT1*, *ESRRA*, *ESRRG*, *GABPB1*, *PPARGC1B*, *PRKAA1*, *PRKAA2*, *TFAM*, or *UCP3* (*p* > 0.05).

### 3.2 Meta‐analysis of *EPAS1* transcriptomic response to aerobic versus resistance training using publicly available datasets

**FIGURE 4:**
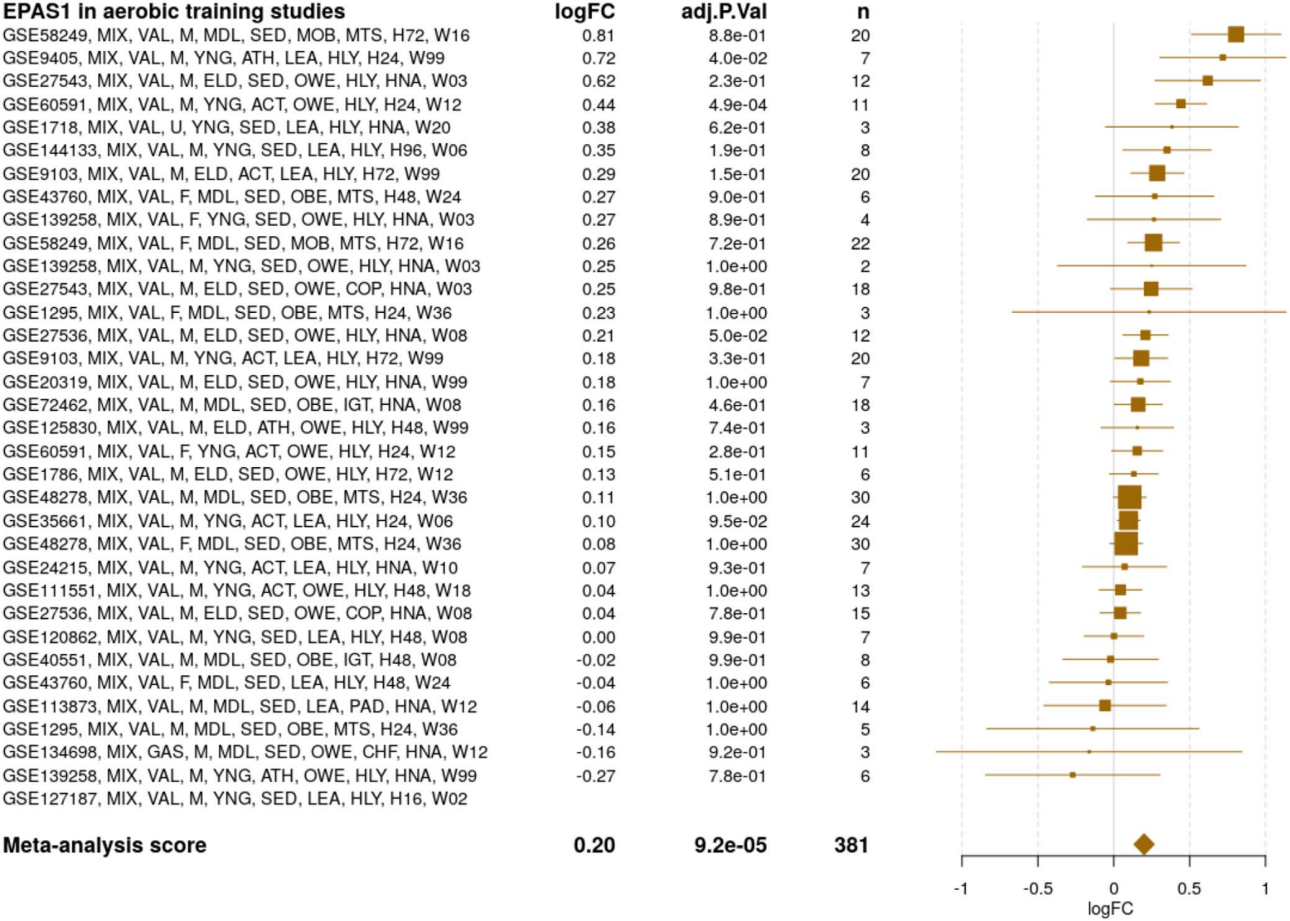
*EPAS1* gene expression in skeletal muscle following aerobic training (*n* = 381, 24 cohorts; *p* = 9.2 × 10^−5^) (MetaMEx, 2025; Pillon et al., 2020).

Analysis of publicly available skeletal muscle transcriptomic datasets (Pillon et al., 2020) showed that *EPAS1* gene expression increased significantly (*p* = 9.2 × 10^−5^; *n* = 381, 24 cohorts) after aerobic training lasting at least 3 weeks (Figure 4), but not after resistance training (*p* > 0.05; *n* = 309, 16 cohorts) (Figure S1) or acute aerobic exercise (*p* > 0.05; *n* = 378, 17 cohorts) (Figure S2). Notably, *EPAS1* was ranked among the top 10 most responsive genes exhibiting increased expression (i.e., among the top positively regulated genes) in response to aerobic training, out of 18,928 genes with available data (Pillon et al., 2020). Stratified analyses showed that the aerobic training–associated increase in *EPAS1* gene expression remained statistically significant in males (*p* = 0.038; *n* = 296), older adults (*p* = 0.037; *n* = 93), and overweight or obese individuals (*p* = 0.026; *n* = 265). In contrast, no statistically significant changes in *EPAS1* expression were observed in females, younger participants, and lean individuals, likely due to limited sample sizes within these subgroups, although the direction of effect remained positive and indicative of a consistent trend.

### 3.3 Genetic association of *EPAS1* rs6756667 with endurance athlete status and aerobic phenotypes: Findings from an original athlete cohort

**FIGURE 5:**
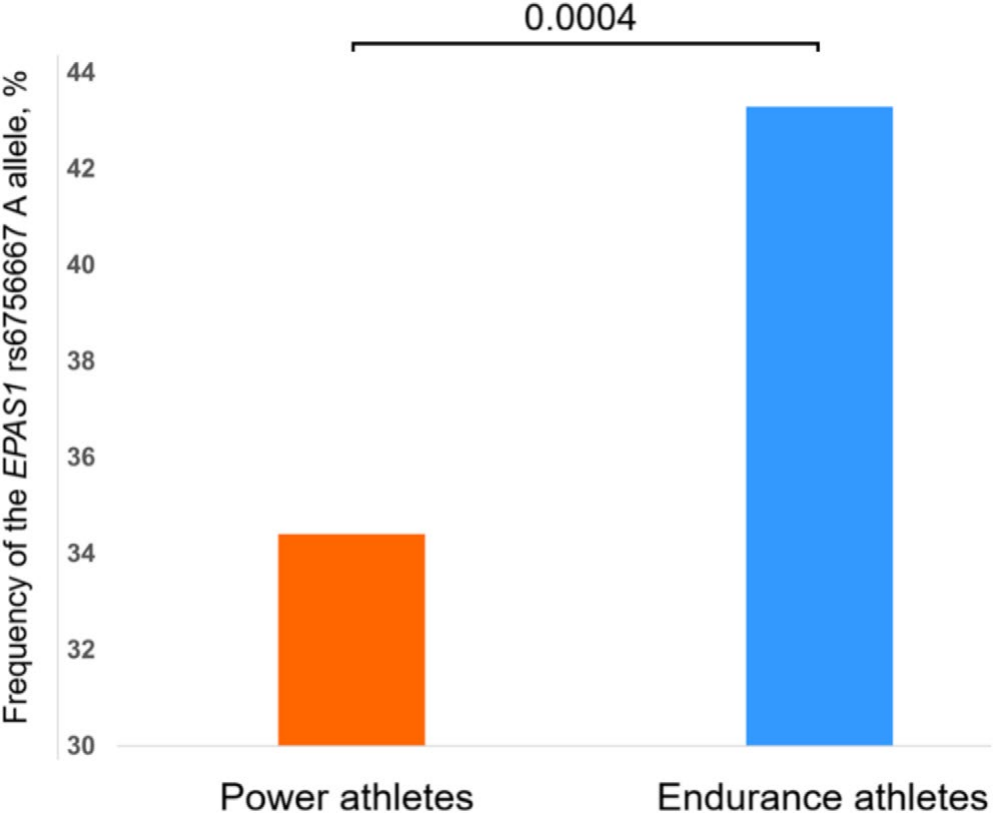
Comparison of the *EPAS1* rs6756667 A allele frequency between power and endurance athletes (OR = 1.5, *p* = 0.0004).

The A allele and the AA genotype of rs6756667 were used as genetic proxies for increased *EPAS1* gene expression (Võsa et al., 2021). In total, 744 athletes (398 endurance and 346 power athletes) were included in the genetic case–control study. The distribution of *EPAS1* rs6756667 genotypes in both endurance (*p* = 0.9616, χ^2^ = 0.002) and power (*p* = 0.6215, χ^2^ = 0.244) athletes was consistent with Hardy–Weinberg equilibrium. Among endurance athletes, the rs6756667 genotype frequencies were 32.2% for GG, 49.0% for AG, and 18.8% for AA, whereas among power athletes, the frequencies were 43.7% for GG, 43.9% for AG, and 12.4% for AA (*p* = 0.0021, χ^2^ = 12.3, comparing the distribution of all genotypes). The frequency of the rs6756667 A allele was significantly higher in endurance athletes than in power athletes (43.3% vs. 34.4%; odds ratio (OR) = 1.5, *p* = 0.0004) (Figure 5). These allelic differences remained significant when endurance and power athletes were analyzed separately in females (A allele: 42.9% vs. 32.5%; OR = 1.6, *p* = 0.0084) and males (A allele: 43.7% vs. 35.6%; OR = 1.4, *p* = 0.015) (Table 2), and after correction for multiple testing (Bonferroni‐adjusted significance threshold: *p* < 0.0166).

**TABLE 2:** Distribution of *EPAS1* rs6756667 genotypes and A allele frequency in endurance and power athletes.

Group	*n*	Genotypes			A allele, %	*p*
		AA	AG	GG		
Female endurance athletes	169	35	75	59	42.9	0.0084*
Male endurance athletes	229	40	120	69	43.7	0.015*
All endurance athletes	398	75	195	128	43.3	0.0004*
Female power athletes	137	14	61	62	32.5	–
Male power athletes	209	29	91	89	35.6	–
All power athletes	346	43	152	151	34.4	–

* *p* < 0.05 indicates statistically significant differences between endurance and power athletes across three comparisons: all athletes, females only, and males only.

**FIGURE 6:**
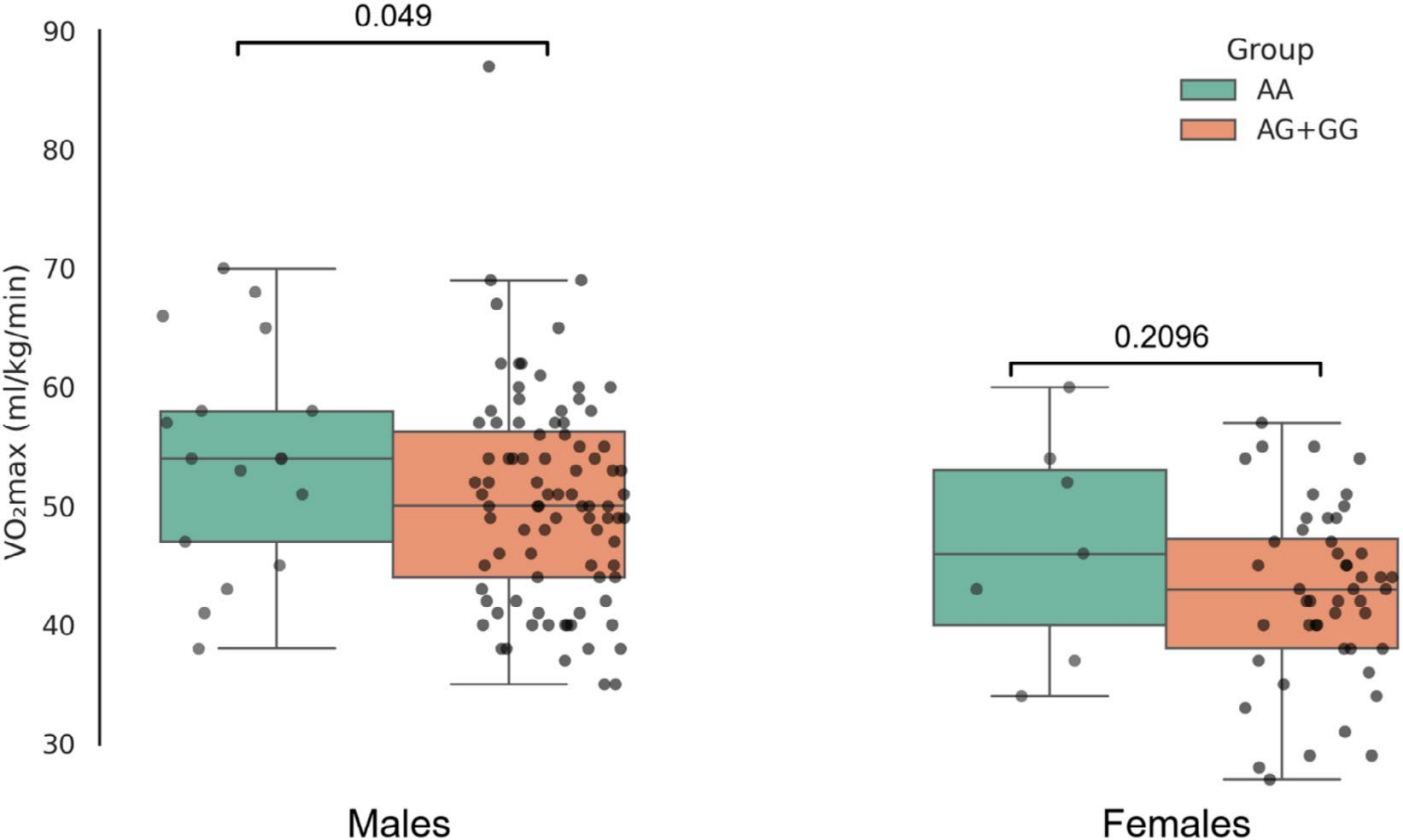
VO_2_max in male (*p* = 0.046) and female (*p* = 0.2096) athletes according to the *EPAS1* rs6756667 genotype (AA vs. AG+GG).

Male athletes with the *EPAS1* rs6756667 AA genotype exhibited significantly higher VO_2_max compared with carriers of the G allele (AA—55.1 ± 9.8 mL/min/kg, AG/GG—50.4 ± 8.7 mL/min/kg; *p* = 0.046). In female athletes, those with the AA genotype tended to have higher VO_2_max than G allele carriers, although the difference did not reach statistical significance (AA—46.6 ± 9.4 mL/min/kg, AG/GG—42.6 ± 7.4 mL/min/kg; *p* = 0.2096) (Figure 6). Across the combined cohort of athletes (*n* = 160), the rs6756667 AA genotype remained associated with higher VO_2_max after adjusting for age, sex, training type, and level of athletic achievement (*p* = 0.02).

**FIGURE 7:**
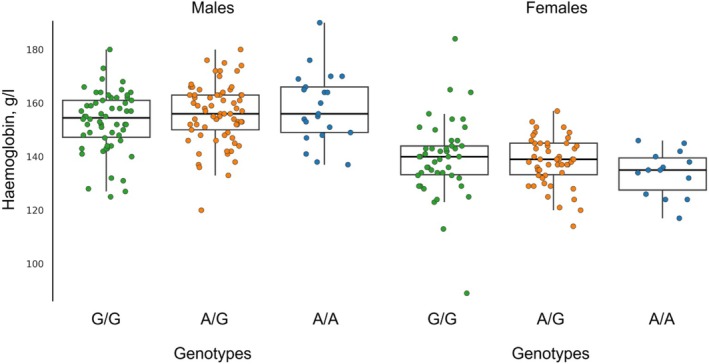
Hemoglobin levels in male and female athletes according to *EPAS1* rs6756667 genotype. *p* Values were obtained from age‐adjusted multiple regression analyses (males: *p* = 0.041; females: *p* = 0.164).

Finally, the *EPAS1* rs6756667 A allele was associated with higher hemoglobin levels in 148 male athletes after adjustment for age (AA—158.2 (13.2) g/L, AG—156.1 (11.1) g/L, GG—153.2 (11.3) g/L; *p* = 0.041), but not in 110 females (AA—133.9 (8.5) g/L, AG—138.3 (8.9) g/L, GG—139.5 (14.3) g/L; *p* = 0.164) (Figure 7).

## 4 DISCUSSION

The present study provides converging transcriptomic, training‐response, and genetic evidence supporting associations between *EPAS1* and human endurance‐related phenotypes. By integrating skeletal muscle gene expression data, large‐scale training transcriptomic datasets, and genotype–phenotype associations in athletes, the findings extend current understanding of *EPAS1* beyond high‐altitude adaptation (Chen et al., 2025; Guo et al., 2015; Shen et al., 2020; Zhang et al., 2020) and highlight its relevance to aerobic performance under normoxic conditions.

Higher *EPAS1* expression in the vastus lateralis of endurance compared with power athletes is consistent with the metabolic and functional demands of prolonged aerobic exercise and aligns with both genetic and transcriptomic evidence associating *EPAS1* with endurance‐oriented phenotypes. At the genetic level, the rs6756667 A allele, used as a proxy for increased *EPAS1* expression (Võsa et al., 2021), was significantly overrepresented in endurance athletes compared with power athletes, an association that remained robust across sexes and after correction for multiple testing. This finding corroborates and substantially extends earlier pilot observations (Jiang et al., 2016) and is consistent with population‐genetic evidence demonstrating strong positive selection at the *EPAS1* locus in high‐altitude populations (Basang et al., 2015). Interestingly, the rs6756667 A allele has also been associated with higher levels of moderate physical activity (*p* = 0.0036) in 343,943 UK Biobank participants (Open Targets Platform, 2025), suggesting that genetically influenced variation in *EPAS1* may be linked to both elite endurance performance and habitual aerobic activity in the general population.

The observed association between the rs6756667 AA genotype and higher VO_2_max in athletes further supports a potential link between *EPAS1* variation and aerobic capacity. Oxygen delivery and utilization are major determinants of VO_2_max (Bassett Jr & Howley, 2000), and EPAS1 regulates several downstream pathways involved in erythropoiesis, vascular function, and mitochondrial metabolism (Moon et al., 2022; Rankin et al., 2007; Takeda et al., 2004). In addition, the association between the rs6756667 A allele and higher hemoglobin levels in male athletes is consistent with the established role of *EPAS1* in erythropoietin regulation and red blood cell homeostasis (Rankin et al., 2007). Enhanced oxygen‐carrying capacity may represent a mechanism through which *EPAS1* variation is associated with improved aerobic performance, although causality cannot be confirmed. The absence of a corresponding association in females may reflect sex‐specific regulation of erythropoiesis or differences in iron status and hormonal milieu. Together, these findings suggest that EPAS1 may support endurance performance by enhancing systemic oxygen transport and facilitating oxygen delivery to working skeletal muscle during prolonged exercise.

In parallel, the meta‐analysis of publicly available transcriptomic datasets (MetaMEx, 2025; Pillon et al., 2020) demonstrated that aerobic, but not resistance, training is associated with increased skeletal muscle *EPAS1* expression. This training specificity reflects the physiological context in which hypoxia‐related signaling is most relevant, namely repeated or sustained reductions in muscle oxygen tension during endurance exercise, and is consistent with reports of increased EPAS1 protein levels following exhaustive exercise in mice (Baker & Parise, 2016), as well as elevated *EPAS1* gene expression after ultra‐endurance events in professional athletes (Maqueda et al., 2017). Importantly, meta‐analytic evidence from multiple independent transcriptomic cohorts indicates that *EPAS1* is preferentially responsive to chronic aerobic exercise, with a consistent upregulation observed after training interventions but not following acute exercise bouts or resistance training. This pattern is further reinforced by its higher basal expression in endurance compared with power athletes, supporting a role for *EPAS1* in long‐term aerobic adaptation rather than transient exercise responses. However, heterogeneity in the populations and training protocols included precludes definitive conclusions regarding causality.

The observed association between *EPAS1* gene expression and the proportion of slow‐twitch muscle fibers in untrained individuals provides additional, albeit moderate, support for a link between EPAS1 and an oxidative muscle phenotype. Although statistically significant, the association exhibited considerable scatter, consistent with the known limitations of the transcriptomic proxy for fiber‐type composition. Moreover, the moderate strength of this association is also likely attributable to the polygenic nature of muscle fiber composition, which is influenced by a large number of genes in addition to *EPAS1* (Schiaffino & Reggiani, 2011). This observation aligns with experimental evidence from animal models showing that EPAS1 acts downstream of PGC‐1α to regulate the slow‐twitch muscle fiber programme (Rasbach et al., 2010), suggesting partial conservation of this regulatory axis in humans, though causality remains untested. Through this pathway, EPAS1 may be indirectly involved in the promotion of oxidative fiber‐type specification, mitochondrial biogenesis, and fatigue resistance, all of which are central determinants of endurance capacity.

The strong positive associations between *EPAS1* expression and multiple endurance‐related genes indicate coordinated transcriptional relationships rather than direct regulatory effects. Notably, most of these genes, including *CKM* (Bouchard et al., 1989; Fedotovskaia et al., 2012), *EPOR* (Nijholt et al., 2021), *HIF1A* (Döring et al., 2010; Gabbasov et al., 2013), *KDR* (*VEGFR2*) (Ahmetov, Hakimullina, et al., 2009; Eider et al., 2013), *MYBPC3* (Al‐Khelaifi et al., 2020), *NFATC4* (Ahmetov, Williams, et al., 2009), *NOS3* (Ahmetov et al., 2008; Varillas‐Delgado et al., 2022), *PPARA* (Ahmetov, Williams, et al., 2009; Akhmetov, Popov, et al., 2007), *PPARD* (Akhmetov, Astranenkova, & Rogozkin, 2007; Wang et al., 2004), *PPARGC1A* (Akhmetov, Popov, et al., 2007; Lin et al., 2002), *UCP2* (Ahmetov et al., 2008), and *VEGFA* (Ahmetov, Williams, et al., 2009; Prior et al., 2006), play established roles in endurance capacity, aerobic adaptation, athletic performance, and slow‐twitch muscle phenotype, reinforcing the biological plausibility of EPAS1 as a central node within endurance‐related transcriptional networks. This hypothesis‐driven approach was adopted to enable focused evaluation of biologically plausible transcriptional relationships between EPAS1 and established endurance‐related molecular pathways, while limiting multiple‐testing burden. Although unbiased genome‐wide correlation and pathway enrichment analyses represent a complementary strategy, the present targeted analysis was designed to prioritize mechanistically interpretable associations within well‐characterized endurance‐relevant networks.

From a mechanistic perspective, these coordinated expression patterns are consistent with a model in which EPAS1 functions as a transcriptional integrator of hypoxia‐sensitive and metabolic signaling pathways, thereby linking local oxygen availability to angiogenic, mitochondrial, and oxidative metabolic adaptations in skeletal muscle. This coordinated co‐expression may be explained, at least in part, by the presence of hypoxia‐response elements (HREs) within the promoters and enhancers of more than 100 hypoxia‐regulated genes, enabling direct transcriptional regulation by HIF complexes, including EPAS1 (Ke & Costa, 2006). In addition, indirect mechanisms may contribute, such as EPAS1‐mediated activation of upstream transcriptional regulators (e.g., PGC‐1α and PPAR signaling pathways), epigenetic chromatin remodeling in response to hypoxic stress, shared regulation by oxygen‐sensitive microRNAs, and fiber type–specific transcriptional programmes characteristic of oxidative skeletal muscle (Rasbach et al., 2010). Through these combined mechanisms, EPAS1 may facilitate long‐term structural and metabolic remodeling of skeletal muscle in response to repeated endurance exercise stimuli, thereby supporting sustained aerobic performance.

We also found that, in untrained subjects, *EPAS1* expression in skeletal muscle was negatively associated with smoking status. This finding is consistent with previous evidence demonstrating decreased *Epas1* gene expression in mice chronically exposed to cigarette smoke (Yoo et al., 2015) and directly addresses one of the aims stated in the study hypothesis. Given the central role of EPAS1 in regulating angiogenesis, mitochondrial function, and oxygen utilization, smoking‐induced downregulation of *EPAS1* may contribute to impaired oxidative capacity and reduced endurance performance, providing a plausible molecular link between cigarette smoking and diminished cardiorespiratory fitness (Zeiher et al., 2019).

Collectively, these findings position EPAS1 as a biologically plausible candidate gene contributing to endurance performance through its associations with muscle fiber composition, endurance‐related gene networks, training responsiveness, and oxygen transport capacity. Rather than acting through a single pathway, EPAS1 is likely to influence endurance capacity via the integrated regulation of multiple physiological systems, including oxygen transport, angiogenesis, mitochondrial metabolism, and fiber‐type composition.

Importantly, the use of a well‐characterized eQTL as a genetic proxy for *EPAS1* expression strengthens causal inference and reduces reliance on purely associative genetic markers. While whole‐genome genotyping and whole‐genome sequencing are becoming increasingly widely used in exercise and sports genetics research (Boulygina et al., 2020; Wang et al., 2025), the integration of functional genomic information, such as eQTL‐based proxies for gene expression, remains essential for improving biological interpretation and highlighting potential mechanisms without over‐interpreting the directionality of effects.

Nevertheless, several limitations should be acknowledged. Skeletal muscle expression data in athletes were limited to males, and therefore conclusions regarding *EPAS1* expression in trained populations apply specifically to male athletes, restricting inference about sex‐specific effects in this group. Detailed information on altitude training history was not available for the participants, and thus we cannot exclude the possibility that hypoxic exposure contributed to the observed elevation of *EPAS1* expression in endurance athletes. Although athletes were instructed to refrain from training for at least 24 h prior to biopsy to minimize acute exercise‐induced transcriptional responses, we acknowledge that this restriction does not completely eliminate the possibility of residual effects from prior training sessions, particularly given the known exercise‐responsive nature of *EPAS1*. Furthermore, while the rs6756667 variant is a robust predictor of *EPAS1* expression in blood, tissue‐specific regulatory effects in skeletal muscle cannot be excluded, and genotype–expression interpretations should therefore be considered with caution. Nevertheless, it is plausible that rs6756667 may influence endurance performance not only via skeletal muscle but also through other tissues, given that *EPAS1* is ubiquitously expressed across human tissues, with particularly high levels in the lungs, vascular endothelial cells, and heart. In addition, fiber‐type composition in the untrained cohort was estimated using a transcriptomic proxy based on mRNA expression of the three *MYH* genes. Although this method provides a reliable approximation, as demonstrated by a strong correlation with immunohistochemically measured fiber proportions in a validation sample of 24 male athletes, it remains an indirect measure and does not fully replace gold‐standard histochemical or immunohistochemical assessments. Importantly, the present study is observational and does not aim to infer causality; all interpretations are therefore restricted to associations and correlations, and no causal claims are made. Finally, although a meta‐analysis of publicly available skeletal muscle transcriptomic datasets (MetaMEx, 2025; Pillon et al., 2020) supports increased *EPAS1* expression following aerobic training, the considerable heterogeneity in the included populations (sex, age, training status, health status, muscle group, and body composition) together with variability in training protocols preclude definitive causal conclusions. Similarly, observed associations between *EPAS1* expression and endurance‐related genes should be interpreted as correlational rather than demonstrating direct regulatory or mechanistic effects. These results are therefore consistent with, but do not prove, a training‐induced effect on *EPAS1* expression or a causal role in the regulation of endurance‐related genes. Future studies incorporating muscle‐specific eQTL data, longitudinal training interventions, and multi‐omics approaches will be valuable in further elucidating the role of EPAS1 in human exercise adaptation.

## 5 CONCLUSION

In summary, the present study demonstrates that EPAS1 is upregulated in endurance‐trained male skeletal muscle, responds to aerobic training, and is genetically associated with endurance athlete status. For the first time in humans, we show that EPAS1 expression is linked to skeletal muscle fiber composition, aerobic capacity, hemoglobin levels in males, and the coordinated regulation of multiple endurance‐related genes. These findings provide novel evidence that EPAS1 contributes to endurance capacity and elite athletic performance.

## AUTHOR CONTRIBUTIONS

**Albina Z. Dautova:** Conceptualization; formal analysis; investigation; supervision. **Elena V. Valeeva:** Formal analysis; investigation. **Ekaterina A. Semenova:** Formal analysis; investigation. **Fanis A. Mavliev:** Formal analysis; investigation. **Alexey A. Zverev:** Investigation; supervision. **Andrey S. Nazarenko:** Conceptualization; formal analysis; investigation; supervision. **George John:** Formal analysis. **Andrey V. Zhelankin:** Formal analysis; investigation. **Andrey K. Larin:** Investigation. **Nikolay A. Kulemin:** Formal analysis. **Rinat I. Sultanov:** Formal analysis; investigation. **Edward V. Generozov:** Conceptualization; funding acquisition; investigation; supervision. **Ildus I. Ahmetov:** Conceptualization; formal analysis; investigation; supervision.

## FUNDING INFORMATION

This study was supported by the Russian Science Foundation (grant no. 24‐15‐00413, “Evaluation of environmental and molecular genetic factors influencing long‐term and short‐term changes in human skeletal muscle fibre phenotype and body composition with regard to physical activity parameters”) and by a state assignment from the Ministry of Sport of the Russian Federation (No. 777‐00010‐25‐00; registration number 1023033100582‐2, “Development of scientifically grounded proposals for enhancing aerobic endurance in athletes, taking genetic determinants into account”).

## CONFLICT OF INTEREST STATEMENT

The authors declare no competing interests.

## ETHICS STATEMENT

This study was approved by the Bioethical Committee of the Volga Region State University of Physical Culture, Sports and Tourism (reference #2; approval date: 26 May 2023) and the Ethics Committee of the Federal Research and Clinical Center of Physical–Chemical Medicine of the Federal Medical and Biological Agency of Russia (reference #2017/04; approval date: 4 July 2017).

## PATIENT CONSENT STATEMENT

Written informed consent was obtained from all participants prior to participation in the study, in accordance with the Declaration of Helsinki and ethical standards for sport and exercise science research. For athletes younger than 18 years, consent was provided by their legal guardians in accordance with Russian legislation.

## Supporting information


Appendix S1.

## Data Availability

RNA‐seq data from athletes and untrained individuals have been deposited in GEO (accession: GSE200398) and dbGaP (accession: phs001048.v2.p1), respectively.
