# Supplementary material for: EPAS1 is associated with human muscle fiber composition and endurance phenotypes
Source: Physiol Rep. 2026 May 19;14(10):e70928. doi: 10.14814/phy2.70928 (PMC13423621; doi:10.14814/phy2.70928)
Supplement: Supplementary file 1 — Appendix S1. [file PHY2-14-e70928-s001.docx]

***EPAS1* is associated with human muscle fibre composition and endurance phenotypes**

**Supplementary file**

**Table S1.** List of a priori selected endurance-related genes and the key references supporting their role in endurance performance, oxidative metabolism, angiogenesis, oxygen transport, muscle fibre specification, and hypoxia-responsive signalling.

| **Gene symbol** | **Protein** | **Function / Associated phenotypes** | **References** |
| --- | --- | --- | --- |
| *AMPD1* | Adenosine monophosphate deaminase 1 | Catalyses the deamination of AMP to IMP + NH₃ in skeletal muscle, regulating adenine nucleotide pool and energy metabolism during contraction. Regulates skeletal muscle ATP catabolism during intense contraction; polymorphism associated with endurance athlete status. | Norman et al. 1998; Macak et al. 2025 |
| *ANGPT1* | Angiopoietin 1 | Secreted glycoprotein that binds Tie2 receptor; stabilises blood vessels and promotes angiogenesis. Promotes angiogenesis and vascular stability in skeletal muscle; part of hypoxia-responsive pathways supporting oxygen delivery during endurance exercise. | Asahara et al. 1998; Kim et al. 2000 |
| *ANGPT2* | Angiopoietin 2 | Secreted glycoprotein; destabilises vessels in absence of VEGF but promotes angiogenesis in presence of VEGF. Promotes angiogenesis in skeletal muscle under hypoxic/endurance conditions. | Mofarrahi et al. 2011; Hoier et al. 2012 |
| *CKM* | Creatine kinase, M-type | Muscle-specific isoform of creatine kinase; catalyses reversible transfer of phosphate between ATP and phosphocreatine. Central to rapid ATP regeneration during muscle contraction; highly expressed in skeletal muscle; associated with energy buffering in endurance exercise. | Bouchard et al. 1989; Fedotovskaia et al. 2012 |
| *CPT1B* | Carnitine palmitoyltransferase 1B | Rate-limiting enzyme for mitochondrial long-chain fatty acid β-oxidation. Facilitates fatty-acid transport into mitochondria for oxidative metabolism; upregulated in endurance-trained muscle; supports aerobic energy production. | Bonnefont et al. 2004; Zeibig et al. 2005 |
| *EPOR* | Erythropoietin receptor | Transmembrane receptor for erythropoietin. Regulates erythropoiesis and oxygen delivery; expressed in skeletal muscle; linked to endurance capacity via improved systemic oxygen transport. | de la Chapelle et al. 1993; Nijholt et al. 2021 |
| *ESRRA* | Estrogen related receptor alpha | Orphan nuclear receptor; transcriptional regulator of mitochondrial and oxidative genes. Drives mitochondrial biogenesis and oxidative metabolism; promotes slow-oxidative phenotype in muscle. | LaBarge et al. 2014; Xia et al. 2023 |
| *ESRRG* | Estrogen related receptor gamma | Orphan nuclear receptor; transcriptional regulator of mitochondrial genes. Regulates mitochondrial biogenesis and oxidative capacity in muscle. Plays an important role in causing a shift toward slow twitch muscle type, and a greater capacity for endurance exercise. | 1. Rangwala et al. 2010; Fan et al. 2025 |
| *FNDC5* | Fibronectin type III domain containing 5 (irisin precursor) | Membrane protein cleaved to irisin; myokine. Irisin promotes browning of adipose tissue and mitochondrial biogenesis in muscle; linked to exercise-induced metabolic adaptation. | Boström et al. 2012; Lavi et al. 2022 |
| *GABPB1* | GA binding protein transcription factor subunit beta 1 | Subunit of GABP (NRF2) transcription factor; regulates nuclear genes for mitochondrial function. Regulates mitochondrial biogenesis and respiratory chain genes; important for oxidative capacity; polymorphism associated with endurance athlete status. | He et al. 2007; Maciejewska-Karłowska et al. 2012 |
| *HIF1A* | Hypoxia inducible factor 1 subunit alpha | Master transcription factor stabilised under hypoxia; activates >100 hypoxia-response genes. Central regulator of hypoxia signalling, angiogenesis (via VEGF) and metabolic adaptation in skeletal muscle; upregulated by endurance exercise; polymorphism associated with aerobic capacity. | Mason et al. 2007; Döring et al. 2010 |
| *KDR* | Kinase insert domain receptor (VEGFR2) | Receptor tyrosine kinase for VEGF; primary mediator of VEGF signalling. Mediates VEGF-induced angiogenesis and vascular permeability in skeletal muscle; critical for capillary density in endurance training; polymorphism associated with endurance athlete status, aerobic capacity and slow-twitch muscle fibres. | Kivelä et al. 2008; Ahmetov et al. 2009; Eider et al. 2013 |
| *MYBPC3* | Myosin binding protein C3 | Regulatory protein that modulates myosin-actin interaction in sarcomere. Regulates cardiac and skeletal muscle contraction kinetics; polymorphism associated with endurance athlete status and aerobic capacity. | Al-Khelaifi et al. 2020 |
| *NFATC4* | Nuclear factor of activated T cells 4 | Calcium-regulated transcription factor; downstream of calcineurin. Regulates mitochondrial energy metabolism; polymorphism associated with endurance athlete status and aerobic capacity. | Bushdid et al. 2003; Ahmetov et al. 2009 |
| *NOS3* | Nitric oxide synthase 3 (endothelial) | Produces nitric oxide from L-arginine in endothelium and muscle. Regulates vascular tone, blood flow and mitochondrial respiration; polymorphism associated with endurance athlete status. | Ahmetov et al. 2008; Lee-Young et al. 2010; Varillas-Delgado et al. 2022 |
| *PPARA* | Peroxisome proliferator activated receptor alpha | Nuclear receptor; transcription factor for fatty-acid oxidation genes. Regulates genes for mitochondrial fatty-acid β-oxidation; supports aerobic metabolism in endurance exercise; polymorphism associated with endurance athlete status, aerobic capacity and slow-twitch muscle fibres. | Akhmetov et al. 2007; Ahmetov et al. 2009; Sabaratnam et al. 2019 |
| *PPARD* | Peroxisome proliferator activated receptor delta | Nuclear receptor; master regulator of fatty-acid catabolism and mitochondrial biogenesis. Strongly promotes slow-twitch fibre proportion, mitochondrial biogenesis and running endurance in transgenic models; polymorphism associated with endurance athlete status. | Wang et al. 2004; Akhmetov et al. 2007 |
| *PPARGC1A* | Peroxisome proliferator activated receptor gamma coactivator 1 alpha (PGC-1α) | Transcriptional coactivator; master regulator of mitochondrial biogenesis. Drives mitochondrial biogenesis, slow-twitch fibre programme and endurance capacity; overexpression increases type I fibres and exercise performance; polymorphism associated with endurance athlete status and aerobic capacity. | Lin et al. 2002; Akhmetov et al. 2007 |
| *PPARGC1B* | Peroxisome proliferator activated receptor gamma coactivator 1 beta | Transcriptional coactivator; regulates mitochondrial biogenesis and angiogenesis in skeletal muscle; polymorphism associated with endurance athlete status and aerobic capacity. | Rowe et al. 2011; Ahmetov et al. 2009 |
| *PRKAA1* | Protein kinase AMP-activated catalytic subunit alpha 1 | Catalytic α1 subunit of AMP-activated protein kinase (AMPK). Energy sensor; activates catabolic pathways and mitochondrial biogenesis during endurance exercise; fibre-type specific expression. | Wu et al. 2018; Coqueiro et al. 2019 |
| *PRKAA2* | Protein kinase AMP-activated catalytic subunit alpha 2 | Catalytic α2 subunit of AMPK. Energy sensor; predominant in skeletal muscle; regulates fatty-acid oxidation and glucose uptake during exercise. | Yu et al. 2003; Coqueiro et al. 2019 |
| *TFAM* | Transcription factor A, mitochondrial | Mitochondrial transcription factor; essential for mtDNA replication and transcription. Regulates mitochondrial DNA copy number and biogenesis; upregulated in endurance training; polymorphism associated with endurance athlete status and aerobic capacity. | Bengtsson et al. 2001;  Ahmetov et al. 2009 |
| *UCP2* | Uncoupling protein 2 | Mitochondrial inner-membrane protein; mild uncoupler of oxidative phosphorylation. Reduces mitochondrial ROS production; modulates energy efficiency in muscle; polymorphism associated with endurance athlete status and aerobic capacity. | Ookawara et al. 2002;  Ahmetov et al. 2008; Ahmetov et al. 2009 |
| *UCP3* | Uncoupling protein 3 | Muscle-specific mitochondrial uncoupler. Protects against lipid-induced mitochondrial damage; associated with fatty-acid metabolism in endurance exercise; polymorphism associated with endurance athlete status and aerobic capacity. | Tsuboyama-Kasaoka et al. 1998; Ahmetov et al. 2009 |
| *VEGFA* | Vascular endothelial growth factor A | Key angiogenic growth factor. Stimulates capillary growth and angiogenesis in skeletal muscle; critical for oxygen delivery during endurance training; upregulated by HIF1A; polymorphism associated with endurance athlete status and aerobic capacity. | Jensen et al. 2004; Prior et al. 2006; Ahmetov et al. 2009 |

**References for Table S1:**

1. Norman B, Mahnke-Zizelman DK, Vallis A, Sabina RL. Genetic and other determinants of AMP deaminase activity in healthy adult skeletal muscle. J Appl Physiol (1985). 1998;85(4):1273-8. doi: 10.1152/jappl.1998.85.4.1273.
2. Macak D, Lee SY, Nyman T, Ampah-Korsah H, Strandback E, Pääbo S, Zeberg H. Muscle AMP deaminase activity was lower in Neandertals than in modern humans. Nat Commun. 2025;16(1):6371. doi: 10.1038/s41467-025-61605-4.
3. Asahara T, Chen D, Takahashi T, Fujikawa K, Kearney M, Magner M, Yancopoulos GD, Isner JM. Tie2 receptor ligands, angiopoietin-1 and angiopoietin-2, modulate VEGF-induced postnatal neovascularization. Circ Res. 1998;83(3):233-40. doi: 10.1161/01.res.83.3.233.
4. Kim I, Kim HG, So JN, Kim JH, Kwak HJ, Koh GY. Angiopoietin-1 regulates endothelial cell survival through the phosphatidylinositol 3'-Kinase/Akt signal transduction pathway. Circ Res. 2000;86(1):24-9. doi: 10.1161/01.res.86.1.24.
5. Mofarrahi M, Hussain SN. Expression and functional roles of angiopoietin-2 in skeletal muscles. PLoS One. 2011;6(7):e22882. doi: 10.1371/journal.pone.0022882.
6. Hoier B, Nordsborg N, Andersen S, Jensen L, Nybo L, Bangsbo J, Hellsten Y. Pro- and anti-angiogenic factors in human skeletal muscle in response to acute exercise and training. J Physiol. 2012;590(3):595-606. doi: 10.1113/jphysiol.2011.216135.
7. Bouchard C, Chagnon M, Thibault MC et al (1989) Muscle genetic variants and trainability. Med Sci Sports Exerc 21:71–77. https://doi.org/10.1249/00005768-198902000-00013
8. Fedotovskaia ON, Popov DV, Vinogradova OL, Akhmetov II (2012) Association of the muscle-specific creatine kinase (CKMM) gene polymorphism with physical performance of athletes. Fiziol Cheloveka 38:105–109
9. Bonnefont JP, Djouadi F, Prip-Buus C, Gobin S, Munnich A, Bastin J. Carnitine palmitoyltransferases 1 and 2: biochemical, molecular and medical aspects. Mol Aspects Med. 2004;25(5-6):495-520. doi: 10.1016/j.mam.2004.06.004.
10. Zeibig J, Karlic H, Lohninger A, Damsgaard R, Smekal G. Do blood cells mimic gene expression profile alterations known to occur in muscular adaptation to endurance training? Eur J Appl Physiol. 2005;95(1):96-104. doi: 10.1007/s00421-005-1334-3.
11. de la Chapelle A, Sistonen P, Lehväslaiho H, Ikkala E, Juvonen E. Familial erythrocytosis genetically linked to erythropoietin receptor gene. Lancet. 1993;341(8837):82-4. doi: 10.1016/0140-6736(93)92558-b.
12. Nijholt KT, Meems LMG, Ruifrok WPT et al (2021) Skeletal muscle erythropoietin receptor and mitochondrial biogenesis. Pflugers Arch 473:1301–1313. https://doi.org/10.1007/s00424-021-02577-4
13. LaBarge S, McDonald M, Smith-Powell L, Auwerx J, Huss JM. Estrogen-related receptor-α (ERRα) deficiency in skeletal muscle impairs regeneration in response to injury. FASEB J. 2014;28(3):1082-97. doi: 10.1096/fj.13-229211.
14. Xia H, Scholtes C, Dufour CR, Guluzian C, Giguère V. ERRα fosters running endurance by driving myofiber aerobic transformation and fuel efficiency. Mol Metab. 2023;78:101814. doi: 10.1016/j.molmet.2023.101814.
15. Rangwala SM, Wang X, Calvo JA, Lindsley L, Zhang Y, Deyneko G, Beaulieu V, Gao J, Turner G, Markovits J. Estrogen-related receptor gamma is a key regulator of muscle mitochondrial activity and oxidative capacity. J Biol Chem. 2010;285(29):22619-29. doi: 10.1074/jbc.M110.125401.
16. Fan W, Oh TG, Wang HJ, Crossley L, He M, Robbins H, Koopari C, Dai Y, Truitt ML, Liddle C, Yu RT, Atkins AR, Downes M, Evans RM. Estrogen-related receptors regulate innate and adaptive muscle mitochondrial energetics through cooperative and distinct actions. Proc Natl Acad Sci U S A. 2025;122(20):e2426179122. doi: 10.1073/pnas.2426179122.
17. Boström P, Wu J, Jedrychowski MP, Korde A, Ye L, Lo JC, Rasbach KA, Boström EA, Choi JH, Long JZ, Kajimura S, Zingaretti MC, Vind BF, Tu H, Cinti S, Højlund K, Gygi SP, Spiegelman BM. A PGC1-α-dependent myokine that drives brown-fat-like development of white fat and thermogenesis. Nature. 2012;481(7382):463-8. doi: 10.1038/nature10777.
18. Lavi G, Horwitz A, Einstein O, Zipori R, Gross O, Birk R. Fndc5/irisin is regulated by myogenesis stage, irisin, muscle type and training. Am J Transl Res. 2022;14(10):7063-7079.
19. He Z, Hu Y, Feng L, Lu Y, Liu G, Xi Y, Wen L, McNaughton LR. NRF2 genotype improves endurance capacity in response to training. Int J Sports Med. 2007;28(9):717-21. doi: 10.1055/s-2007-964913.
20. Maciejewska-Karłowska A, Leońska-Duniec A, Cięszczyk P, Sawczuk M, Eider J, Ficek K, Sawczyn S. The GABPB1 gene A/G polymorphism in Polish rowers. J Hum Kinet. 2012;31:115-20. doi: 10.2478/v10078-012-0012-x.
21. Mason SD, Rundqvist H, Papandreou I, Duh R, McNulty WJ, Howlett RA, Olfert IM, Sundberg CJ, Denko NC, Poellinger L, Johnson RS. HIF-1alpha in endurance training: suppression of oxidative metabolism. Am J Physiol Regul Integr Comp Physiol. 2007;293(5):R2059-69. doi: 10.1152/ajpregu.00335.2007.
22. Döring F, Onur S, Fischer A et al (2010) HIF1A Pro582Ser polymorphism in elite endurance athletes. J Appl Physiol 108:1497–1500. https://doi.org/10.1152/japplphysiol.01165.2009
23. Kivelä R, Silvennoinen M, Lehti M, Jalava S, Vihko V, Kainulainen H. Exercise-induced expression of angiogenic growth factors in skeletal muscle and in capillaries of healthy and diabetic mice. Cardiovasc Diabetol. 2008;7:13. doi: 10.1186/1475-2840-7-13.
24. Ahmetov II, Hakimullina AM, Popov DV et al (2009) VEGFR2 His472Gln polymorphism and endurance phenotypes. Eur J Appl Physiol 107:95–103. https://doi.org/10.1007/s00421-009-1105-7
25. Eider J, Leonska-Duniec A, Maciejewska-Karlowska A et al (2013) VEGFR2 polymorphism in Polish endurance athletes. Int SportMed J 14:29–35
26. Al-Khelaifi F, Yousri NA, Diboun I et al (2020) MYBPC3 polymorphism and endurance athlete status. Front Genet 11:595. https://doi.org/10.3389/fgene.2020.00595
27. Bushdid PB, Osinska H, Waclaw RR, Molkentin JD, Yutzey KE. NFATc3 and NFATc4 are required for cardiac development and mitochondrial function. Circ Res. 2003;92(12):1305-13. doi: 10.1161/01.RES.0000077045.84609.9F.
28. Ahmetov II, Williams AG, Popov DV et al (2009) Combined impact of metabolic gene polymorphisms on endurance status. Hum Genet 126:751–761. https://doi.org/10.1007/s00439-009-0728-4
29. Ahmetov II, Popov DV, Astratenkova IV et al (2008) The use of molecular genetic methods for prognosis of aerobic and anaerobic performance in athletes. Hum Physiol 34:338–342. https://doi.org/10.1134/S0362119708030110
30. Lee-Young RS, Ayala JE, Hunley CF, James FD, Bracy DP, Kang L, Wasserman DH. Endothelial nitric oxide synthase is central to skeletal muscle metabolic regulation and enzymatic signaling during exercise in vivo. Am J Physiol Regul Integr Comp Physiol. 2010;298(5):R1399-408. doi: 10.1152/ajpregu.00004.2010.
31. Varillas-Delgado D, Morencos E, Gutiérrez-Hellín J et al (2022) Genetic profiles to identify endurance talent. PLoS One 17:e0274880. https://doi.org/10.1371/journal.pone.0274880
32. Akhmetov II, Popov DV, Mozhaĭskaia IA et al (2007) Association of regulatory genes polymorphisms with aerobic and anaerobic performance of athletes. Ross Fiziol Zh Im I M Sechenova 93:837–843
33. Sabaratnam R, Pedersen AJ, Eskildsen TV, Kristensen JM, Wojtaszewski JFP, Højlund K. Exercise Induction of Key Transcriptional Regulators of Metabolic Adaptation in Muscle Is Preserved in Type 2 Diabetes. J Clin Endocrinol Metab. 2019;104(10):4909-4920. doi: 10.1210/jc.2018-02679.
34. Wang YX, Zhang CL, Yu RT et al (2004) Regulation of muscle fiber type and endurance by PPARδ. PLoS Biol 2:e294. https://doi.org/10.1371/journal.pbio.0020294
35. Akhmetov II, Astranenkova IV, Rogozkin VA (2007) Association of PPARD gene polymorphism with human physical performance. Mol Biol 41:852–857
36. Lin J, Wu H, Tarr PT et al (2002) PGC-1α drives formation of slow-twitch muscle fibres. Nature 418:797–801. https://doi.org/10.1038/nature00904
37. Rowe GC, Jang C, Patten IS, Arany Z. PGC-1β regulates angiogenesis in skeletal muscle. Am J Physiol Endocrinol Metab. 2011;301(1):E155-63. doi: 10.1152/ajpendo.00681.2010.
38. Wu W, Xu Z, Zhang L, Liu J, Feng J, Wang X, Shan T, Wang Y. Muscle-specific deletion of Prkaa1 enhances skeletal muscle lipid accumulation in mice fed a high-fat diet. J Physiol Biochem. 2018;74(2):195-205. doi: 10.1007/s13105-017-0604-y.
39. Coqueiro RDS, Soares TJ, Pereira R, Correia TML, Coqueiro DSO, Oliveira MV, Marques LM, de Sá CKC, de Magalhães ACM. Therapeutic and preventive effects of exercise on cardiometabolic parameters in aging and obese rats. Clin Nutr ESPEN. 2019;29:203-212. doi: 10.1016/j.clnesp.2018.10.003.
40. Yu M, Stepto NK, Chibalin AV, Fryer LG, Carling D, Krook A, Hawley JA, Zierath JR. Metabolic and mitogenic signal transduction in human skeletal muscle after intense cycling exercise. J Physiol. 2003;546(Pt 2):327-35. doi: 10.1113/jphysiol.2002.034223.
41. Bengtsson J, Gustafsson T, Widegren U, Jansson E, Sundberg CJ. Mitochondrial transcription factor A and respiratory complex IV increase in response to exercise training in humans. Pflugers Arch. 2001;443(1):61-6. doi: 10.1007/s004240100628.
42. Ookawara T, Suzuk K, Haga S, Ha S, Chung KS, Toshinai K, Hamaoka T, Katsumura T, Takemasa T, Mizuno M, Hitomi Y, Kizaki T, Suzuki K, Ohno H. Transcription regulation of gene expression in human skeletal muscle in response to endurance training. Res Commun Mol Pathol Pharmacol. 2002;111(1-4):41-54.
43. Tsuboyama-Kasaoka N, Tsunoda N, Maruyama K, Takahashi M, Kim H, Ikemoto S, Ezaki O. Up-regulation of uncoupling protein 3 (UCP3) mRNA by exercise training and down-regulation of UCP3 by denervation in skeletal muscles. Biochem Biophys Res Commun. 1998;247(2):498-503. doi: 10.1006/bbrc.1998.8818.
44. Jensen L, Pilegaard H, Neufer PD, Hellsten Y. Effect of acute exercise and exercise training on VEGF splice variants in human skeletal muscle. Am J Physiol Regul Integr Comp Physiol. 2004;287(2):R397-402. doi: 10.1152/ajpregu.00071.2004.
45. Prior SJ, Hagberg JM, Paton CM et al (2006) VEGF promoter variation and maximal oxygen consumption. Am J Physiol Heart Circ Physiol 290:H1848–H1855. https://doi.org/10.1152/ajpheart.01033.2005


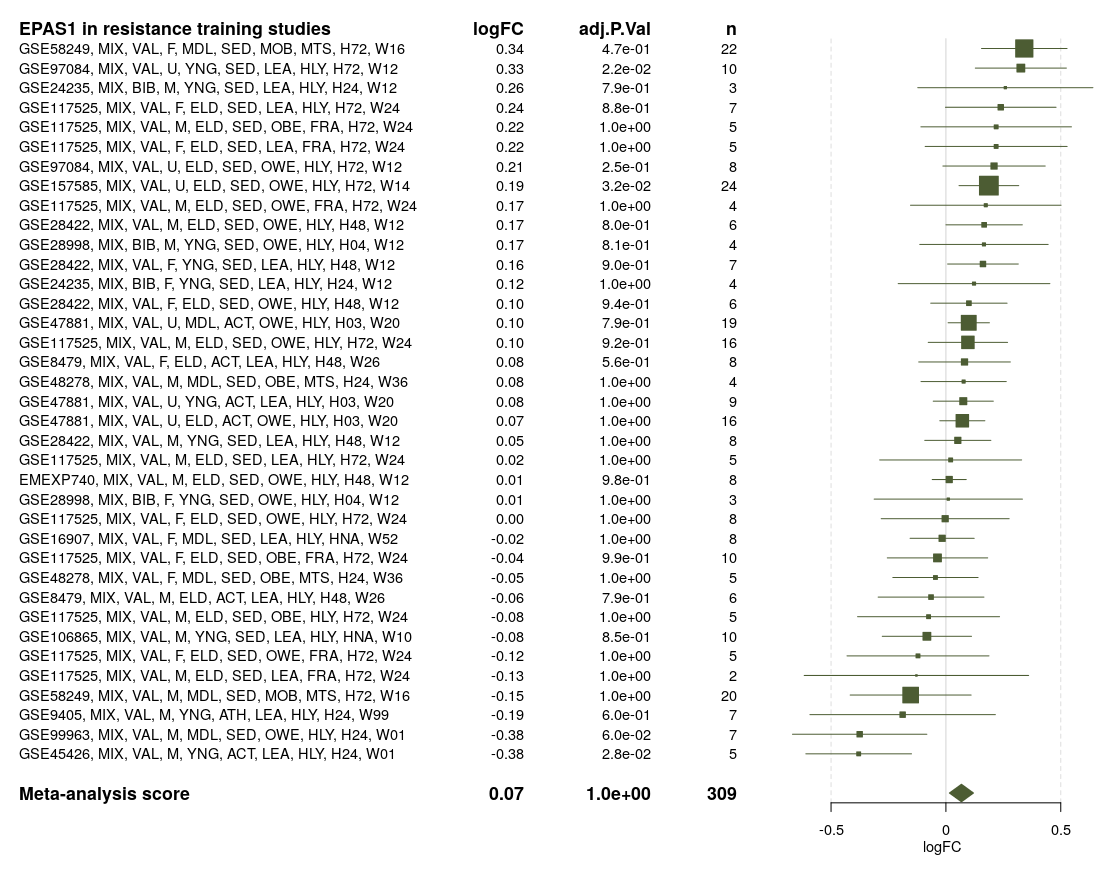


**Figure S1.** *EPAS1* gene expression in skeletal muscle following resistance training (*n* = 309, 16 cohorts; *p* > 0.05) [MetaMEx; Pillon et al. 2020].


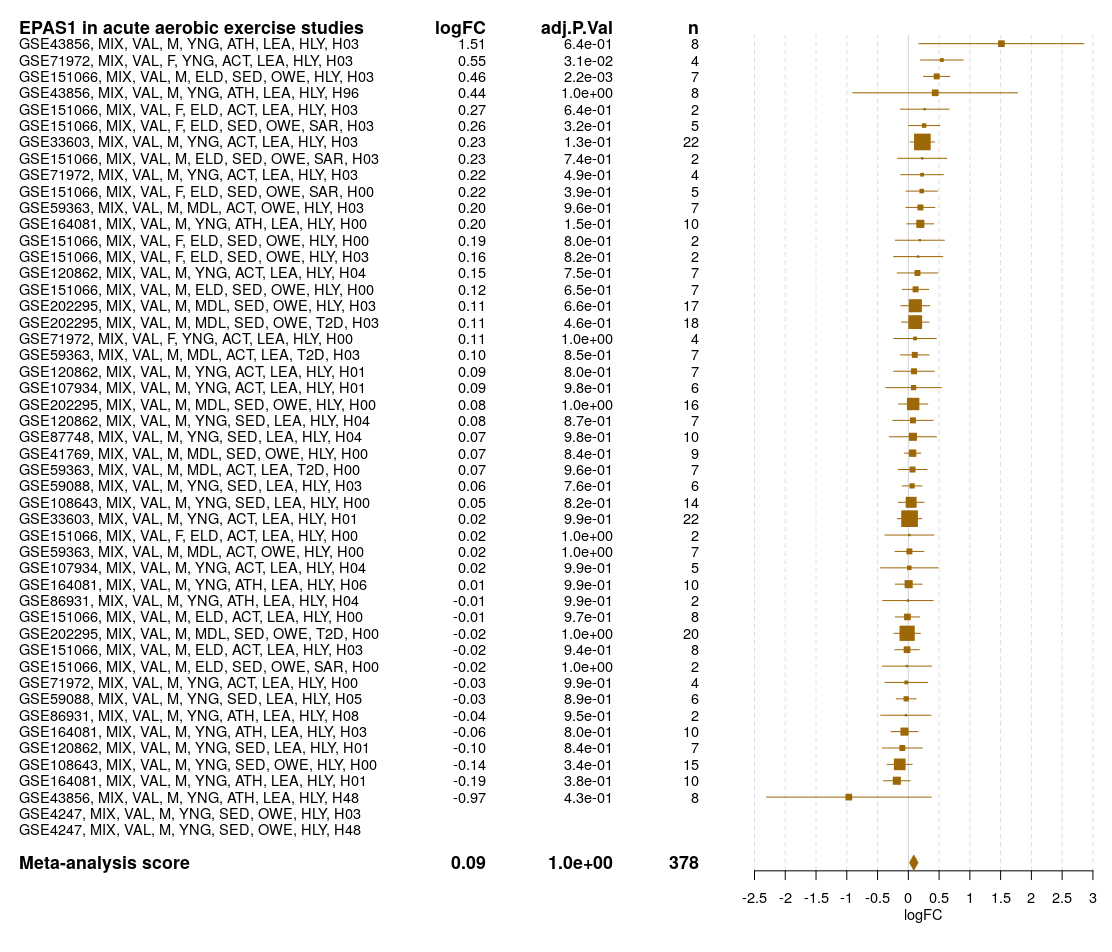


**Figure S2.** *EPAS1* gene expression in skeletal muscle following acute aerobic exercise (*n* = 378, 17 cohorts; *p* > 0.05) [MetaMEx; Pillon et al. 2020].

**References for Figures S1 and S2:**

1. MetaMEx (2025) Accessed 19 April 2026. <https://metamex.eu/app/metamex>
2. Pillon NJ, Gabriel BM, Dollet L et al (2020) Transcriptomic profiling of skeletal muscle adaptations to exercise and inactivity. Nat Commun 11:470. https://doi.org/10.1038/s41467-019-13869-w
